# Risk analysis of depression among adult patients with epilepsy of different sex: a retrospective single-center study from China

**DOI:** 10.3389/fpsyt.2023.1283983

**Published:** 2023-12-04

**Authors:** Wang Guo, Yu-xuan Li, Yi Zhang, Xue-rui Lv, Sheng-xue Wang, Shuang-yuan Zhang, En-si Wang, Xin-jie Chen, Yun Li

**Affiliations:** ^1^Clinical Medical School, Dali University, Dali, China; ^2^Department of Neurology, The First Affiliated Hospital of Dali University, Dali, China

**Keywords:** epilepsy, patients with epilepsy, depression, sex difference, risk factors

## Abstract

**Objective:**

To determine sex differences in the prevalence of depression and assess the risk factors for depression among adult patients with epilepsy from the Dali area of China.

**Methods:**

We retrospectively analyzed the clinical data of adult patients with epilepsy who visited the First Affiliated Hospital of Dali University from January 2017 to January 2022. Patient Health Questionnaire-9 was used to assess depressive symptoms in patients with epilepsy. The risk factors of depression were analyzed by binary logistic regression among different sex in patients with epilepsy.

**Results:**

There were significant sex differences in depression in patients with epilepsy (*p <* 0.001), and females were 4.27 times more likely to suffer from depression than males (95% confidence interval: 3.70–4.92). The risk factors for depression among female patients with epilepsy included occupation (*p <* 0.001), years with epilepsy (*p <* 0.001), seizure frequency (*p <* 0.001), seizure type (*p <* 0.001), etiology (*p <* 0.001), number of antiseizure medications used (*p <* 0.001), antiseizure medications (*p <* 0.001), and electroencephalogram findings (*p <* 0.001). The risk factors for depression among male patients with epilepsy included age (*p <* 0.001), ethnicity (*p <* 0.001), occupation (*p <* 0.001), years with epilepsy (*p <* 0.001), seizure frequency (*p <* 0.001), seizure type (*p <* 0.001), etiology (*p <* 0.001), number of antiseizure medications used (*p* < 0.001), antiseizure medications (*p <* 0.001), and electroencephalogram findings (*p <* 0.001).

**Conclusion:**

Adult female patients with epilepsy had a higher risk of depression than adult male patients with epilepsy. There were sex differences in the risk factors associated with depression among patients with epilepsy.

## 1 Introduction

Epilepsy is a prevalent neurological disorder, affecting approximately 70 million individuals worldwide (1). Epidemiological data has shown that the annual incidence rate of epilepsy is approximately 50.4–81.7/100,000 (2). A 2005 report from the World Health Organization showed that epilepsy accounted for 0.5% of the global health burden (3). Beyond the deleterious effects of epilepsy itself, the presence of associated emotional and psychiatric disorders, including depression, anxiety, and autism spectrum disorder, is gaining growing attention. Patients with epilepsy may suffer heightened psychological stress because of the repetitive nature of epilepsy. This also leads to a higher prevalence of emotional disorders among patients with epilepsy than among the general population and patients with other chronic diseases (4). Moreover, depression, recognized as a mental disorder, is also considered as a common comorbidity linked with epilepsy (5). Depression is frequently seen as anxiety in patients with epilepsy (6). A threefold higher prevalence of depression among patients with epilepsy than among those without epilepsy has been reported (7). The prevalence of depression is approximately 55% in adults with temporal lobe epilepsy and drug-resistant epilepsy (8). In addition, the prevalence of the major depressive disorder is 17.4% (10.0–24.9%) in patients with epilepsy and as high as 20–60% in patients with drug-resistant epilepsy (9, 10). Major depressive disorder remains an important risk factor for suicide in patients with epilepsy (11). In the general population, a higher prevalence of depression has been shown among women than men (12). In patients with epilepsy, some studies have also observed that females exhibit a greater propensity for experiencing depressive symptoms than males (13, 14). In contrast, some studies from China and Ethiopia have found no significant sex differences in epilepsy comorbid depression (15–17). A meta-analysis encompassing diverse populations such as Asian, African, Caucasian, and others, further affirmed the lack of correlation between gender and depression in individuals diagnosed with epilepsy (18). Therefore, further extensive research is imperative to investigate potential sex discrepancies among epilepsy comorbid depression. The risk factors associated with depression in epilepsy patients may exhibit gender-specific variations. Nevertheless, there remains a lack of extensive large-scale studies investigating potential variations in the risk factors for depression among epilepsy patients of diverse genders. Hence, further studies on comorbid depression among patients with epilepsy are warranted, with a specific focus on risk factors for depression of diverse genders.

At present, the research on depression in patients with epilepsy is steadily growing. Lower literacy levels, head trauma, combination therapy with antiseizure medications (ASMs), and focal epilepsy are associated with high anxiety. However, these associations are shown to be not independent (19). A study conducted on risk factors for epilepsy comorbid depression residing in rural Sichuan in China revealed that annual income was associated with risk of depression (20). A cross-sectional study found that the level of education, place of residence, shame, and seizure frequency were significantly associated with depression (21). Many studies have shown that age, sex, marital status, medication dosage, and adverse medication events are also associated with depression in patients with epilepsy (22–26). A study has fund that level of education is a risk factor for depression in male patients with epilepsy, whereas the seizure frequency, epilepsy type, number of ASMs used, age, and settlement are not significantly correlated in females and males (16).

Currently, the risk analysis of depression in patients with epilepsy is gradually increasing, whereas studies on different sex are still lacking in international. Furthermore, the extant large-scale investigations into the association between epilepsy and the risk of depression predominantly emanate from the United Kingdom and the United States. The study from United Kingdom showed that depression was associated with an increased risk of epilepsy, but did not delve into specific risk factors influencing the onset of depression (27). The study by Viguera et al. (28) found that black race and lower income were risk factors for depression among patients with epilepsy, but there was still a lack of more research on risk factors. Significantly, there is still a lack of large-sample studies from China, particularly with regard to comprehensive studies examining the risk to depression in patients with epilepsy across varying sex profiles. Thus, we herein determined sex differences in the prevalence of depression and assessed the risk factors for depression among adult patients with epilepsy from the Dali area of China.

## 2 Materials and methods

### 2.1 Study design

This study is a single-center retrospective study using data from the Epilepsy Center of the First Affiliated Hospital of Dali University. The First Affiliated Hospital of Dali University is a secondary epilepsy center and a teaching hospital of Dali University. This study enrolled patients who had received inpatient or outpatient treatments at our center during January 2017–2022. All patients had undergone psychological testing (Patient Health Questionnaire-9, PHQ-9) upon admission. Inclusion criteria: (1) the diagnosis of epilepsy consistent with the standard of the 2014 International League Against Epilepsy (29), (2) patients aged ≥18 years. Exclusion criteria: (1) patients with cognitive dysfunction (Assessment reliant upon the score obtained from the Mini-Mental State Examination at the time of admission) and psychiatric disorders (e.g., schizophrenia), (2) patients who had not undergone psychological tests at the time of admission. Reconfirmation of epilepsy diagnosis in the included patients was performed based on the clinical history and electroencephalogram (EEG) results. All data and diagnoses were confirmed by two neurologists. The following data were collected from the medical records: the score of PHQ-9, age, sex, ethnicity, occupation, level of educational, marital status, place of residence, years with epilepsy, seizure frequency (within the last 1 year), seizure type, etiology, number of ASMs used, ASMs (Statistical analysis was conducted for patients who used only one type of ASMs), and EEG findings.

PHQ-9 is a short and effective tool used for assessing depressive symptoms. It contains 9 items that reflect the mood of patients in the past 2 weeks (30). Each item contains a typical symptom of depressive disorder, and it is assessed by how often the symptoms have appeared in the past 2 weeks. Each item of this questionnaire consisted of the following 4 answers: “Not at all,” “A few days,” “More than half days,” and “Nearly every day,” which correspond to a score of “0,” “1,” “2,” and “3.” Past studies have shown that its specificity and sensitivity are high and that it has been widely used in the evaluation of epilepsy and depression. PHQ-9 is increasingly being used to evaluate depression in patients with epilepsy, with good applicability. Past studies have demonstrated that the PHQ-9 score of ≥10 best diagnoses depression (31). In this study, the Chinese versions of the PHQ-9 scales are used to evaluate depression in patients with epilepsy. Patients with PHQ-9 scores ≥10 are classified into the depression group. This study is retrospective in nature and do not necessitate the signing of informed consent forms by the patients. The research protocol received approval from the Ethics Committee of the First Affiliated Hospital of Dali University.

### 2.2 Statistical analysis

All patients were categorized by sex and their data were subjected to statistical analysis. Categorical variables were presented as percentages. The Chi-square test or Fisher’s exact test was used to analyze the categorical variables in patients with epilepsy with or without depression. The binary logistic regression model was applied to analyze the risk factors (variables with *p* < 0.05 in univariate analysis) among the depression group in patients with epilepsy.

## 3 Results

### 3.1 Demographic characteristics

Overall, 3,620 eligible patients were included in the present study. Among them, 1,685 (46.55%) patients were female and 1,935 (53.45%) were male. The mean age of females was 36 (standard deviation: 15.05) years and that of males was 39 (standard deviation: 15.83) years. The ethnic distribution was as follows: 1620 (44.75%) Han, 1,113 (30.75%) Bai, 186 (5.14%) Yi, 184 (5.08%) Tibetan, 296 (8.18%) Lisu, and 221 (6.10%) others. Concerning occupation, there were 2,265 (62.57%) farmers, 520 (14.36%) employees, 602 (16.63%) students, and 233 (6.44%) with other jobs. A total of 1725 (47.65%) patients were married and 1895 (52.35%) were unmarried. There were 2048 (56.57%) patients from rural areas and 1,572 (43.43%) from urban areas. The detailed demographic characteristics of patients were shown in Table 1.

**Table 1:** Clinical and demographic characteristics among different sex in patients with epilepsy (*n* = 3,620).

	Female				Male			
Variable	With depression	Without depression	Statistical value	*p*	With depression	Without depression	Statistical value	*p*
Age			10.90	**0.012**			26.75	**<0.001**
18–26	311(32.13%)	207(28.87%)			143(30.75%)	430(29.25%)		
27–45	478(49.38%)	331(46.17%)			215(46.24%)	538(36.60%)		
46–64	143(14.77%)	148(20.64%)			73(15.70%)	394(26.80%)		
≥ 65	36(3.72%)	31(4.32%)			34(7.31%)	108(7.35%)		
Ethnicity			10.10	0.072			77.71	**<0.001**
Han Chinese	395(40.81%)	325(45.33%)			215(46.24%)	685(46.60%)		
Bai	334(34.50%)	251(35.01%)			70(15.05%)	458(31.15%)		
Yi	52(5.37%)	20(2.79%)			42(9.03%)	72(4.90%)		
Tibetan	56(5.79%)	36(5.02%)			26(5.59%)	66(4.49%)		
Lisu	107(11.05%)	72(10.04%)			36(7.74%)	81(5.51%)		
others	24(2.48%)	13(1.81%)			76(16.35%)	108(7.35%)		
Occupation			47.51	**<0.001**			87.37	**<0.001**
Farmer	645(66.63%)	416(58.02%)			365(78.49%)	839(57.07%)		
Employee	72(7.44%)	89(12.41%)			36(7.74%)	323(21.97%)		
Student	143(14.77%)	171(23.85%)			37(7.96%)	251(17.08%)		
Others	108(11.16%)	41(5.72%)			27(5.81%)	57(3.88%)		
Level of education			0.72	0.396			2.65	0.104
Below High School	681(70.35%)	518(72.25%)			358(76.99%)	1,076(73.20%)		
Bachelor degree or above	287(29.65%)	199(27.75%)			107(23.01%)	394(26.80%)		
Marital status			0.69	0.408			2.95	0.086
Married	430(44.42%)	304(42.40%)			222(47.74%)	769(52.31%)		
Unmarried	538(55.58%)	413(57.60%)			243(52.26%)	701(47.69%)		
Place of residence			0.10	0.754			3.13	0.077
Rural	603(62.29%)	452(63.04%)			222(47.74%)	771(52.45%)		
Urban	365(37.71%)	265(36.96%)			243(52.26%)	699(47.55%)		
Years with epilepsy			117.19	**<0.001**			62.00	**<0.001**
< 1 year	288(29.75%)	204(28.45%)			163(35.05%)	645(43.88%)		
1-3 years	107(11.06%)	226(31.52%)			159(34.20%)	251(17.07%)		
> 3 years	573(59.19%)	287(40.03%)			143(30.75%)	574(39.05%)		
Seizure frequency			59.35	**<0.001**			40.37	**<0.001**
Seizure-free	323(33.37%)	179(24.96%)			72(15.48%)	548(37.28%)		
< 1 time per month	137(14.15%)	210(29.29%)			251(53.98%)	466(31.70%)		
≥ 1 time per month	508(52.48%)	328(45.75%)			142(30.54%)	456(31.02%)		
Seizure type			56.90	**<0.001**			360.48	**<0.001**
Focal	430(44.42%)	190(26.50%)			393(84.52%)	502(34.15%)		
Generalized	538(55.58%)	527(73.50%)			72(15.48%)	968(65.85%)		
Etiology			167.14	**<0.001**			220.53	**<0.001**
Unknown	538(55.58%)	589(82.15%)			143(30.75%)	753(51.22%)		
Structural	170(17.56%)	32(4.46%)			108(23.22%)	358(24.35%)		
Infectious	188(19.42%)	36(5.02%)			71(15.27%)	266(18.10%)		
Metabolic	56(5.79%)	47(6.56%)			132(28.39%)	72(4.90%)		
Immune	16(1.65%)	13(1.81%)			11(2.37%)	21(1.43%)		
Number of ASMs used			20.81	**<0.001**			40.36	**<0.001**
0	13(1.34%)	32(4.46%)			33(7.10%)	110(7.48%)		
1	649(67.05%)	502(70.01%)			324(69.68%)	789(53.67%)		
≥ 2	306(31.61%)	183(25.53%)			108(23.22%)	571(38.85%)		
ASMs			142.52	**<0.001**			247.86	**<0.001**
Sodium valproate	72(11.09%)	167(33.27%)			54(16.67%)	297(37.64%)		
Carbamazepine	77(11.86%)	115(22.91%)			43(13.27%)	208(26.36%)		
Lacosamide	52(8.01%)	29(5.78%)			39(12.04%)	69(8.74%)		
Lamotrigine	49(7.55%)	35(6.97%)			32(9.88%)	122(15.47%)		
Levetiracetam	298(45.92%)	104(20.72%)			120(37.04%)	31(3.93%)		
Topiramate	65(10.02%)	21(4.18%)			25(7.71%)	34(4.31%)		
Oxcarbazepine	36(5.55%)	31(6.17%)			11(3.39%)	28(3.55%)		
EEG findings			97.45	**<0.001**			127.74	**<0.001**
Temporal area discharge	341(39.36%)	129(23.57%)			110(23.66%)	387(31.77%)		
Others	139(18.49%)	221(36.40%)			262(56.34%)	354(26.80%)		
No discharge	368(42.15%)	247(40.03%)			93(20.00%)	529(41.43%)		

The chi-square test revealed a significant difference in sex (*p* < 0.001) between patients with epilepsy with and without depression. Females were more likely to be depressed than males (Table 2). Females were 4.27 times more likely to suffer from depression than males [odds ratio (OR) = 4.27, 95% confidence interval (CI): 3.70–4.92]. In univariate analysis, in order to observe whether a single ASM had an effect on depression, we selected subjects who were treated with only one ASM from the “number of ASMs used” group to conduct chi-square test and binary logistic regression analysis. These ASMs included sodium valproate, carbamazepine, lacosamide, lamotrigine, levetiracetam, topiramate, and oxcarbazepine. Univariate analysis revealed significant differences in age (*p =* 0.012), occupation (*p* < 0.001), years with epilepsy (*p* < 0.001), seizure frequency (*p* < 0.001), seizure type (*p* < 0.001), etiology (*p* < 0.001), number of ASMs used (*p* < 0.001), ASMs (*p* < 0.001), and EEG findings (*p* < 0.001) among female patients with epilepsy with and without comorbid depression. However, the ethnicity (*p =* 0.072), level of education (*p =* 0.396), marital status (*p =* 0.408), and place of residence (*p =* 0.754) were not significantly different between female patients with epilepsy with and without depression (Table 1). Univariate analysis revealed significant differences in age (*p* < 0.001), ethnicity (*p* < 0.001), occupation (*p* < 0.001), years with epilepsy (*p* < 0.001), seizure frequency (*p* < 0.001), seizure type (*p* < 0.001), etiology (*p* < 0.001), number of ASMs used (*p* < 0.001), ASMs (*p* < 0.001), and EEG findings (*p* < 0.001) among male patients with epilepsy with and without comorbid depression. However, the level of education (*p =* 0.104), marital status (*p =* 0.086), and place of residence (*p =* 0.077) did not show significant differences among male patients with epilepsy with and without comorbid depression (Table 1).

**Table 2:** The sex characteristics and multivariate logistic regression analysis for sex in patients with epilepsy.

Variable	With depression	Without depression	*χ* ^2^	*p*	a
Sex			420.563	**< 0.001**
Female	968(67.55%)	717(32.78%)		
Male	465(32.45%)	1,470(67.22%)		
b			95% CI	
	OR	*p*	Lower limit	Upper limit
Sex		**< 0.001**		
Female	4.27	**< 0.001**	3.70	4.92
Male	1			

^a^Chi-square tests for sex between those with and without depression in patients with epilepsy. ^b^Binary logistic regression analysis for sex of depression in patients with epilepsy.

### 3.2 Prevalence and severity of depression symptoms in patients with epilepsy

In total, 1,433 (39.59%) patients were considered to have depression (PHQ-9 score ≥ 10), including 968 (26.74%) females and 465 (12.85%) males. A total of 537 patients (14.83%), including 287 females (7.93%) and 250 males (6.90%), appeared PHQ-9 score of ≥20 in our study (Table 3).

**Table 3:** The severity of depression among different sex in patients with epilepsy (*n* = 3,620).

Depression severity	PHQ-7 score	N (%)	
		Female	Male
Minimal or No depression	0–4	681(40.41%)	1,183(61.14%)
Mild depression	5–9	36(2.14%)	287(14.83%)
Moderate depression	10–14	430(25.52%)	179(9.25%)
Moderately severe depression	15–19	251(14.90%)	36(1.86%)
Severe depression	20–27	287(17.03%)	250(12.92%)

### 3.3 Factors influencing depression among adult female patients with epilepsy

Variables with *p* < 0.05 in the univariate analysis were included in the regression analysis, including age, occupation, years with epilepsy, seizure frequency, seizure type, etiology, number of ASMs used, ASMs, and EEG findings (Table 1). Occupation (*p <* 0.001), years with epilepsy (*p <* 0.001), seizure frequency (*p <* 0.001), seizure type (*p <* 0.001), etiology (*p <* 0.001), number of ASMs used (*p <* 0.001), ASMs (*p <* 0.001), and EEG findings (*p <* 0.001) had an independent effect on depression in female patients with epilepsy. Females with epilepsy for over 3 years (OR = 1.41, 95% CI: 1.13–1.78) were more likely to develop depression than those with epilepsy for less than 1 year. Focal epilepsy was more likely to cause depression than generalized epilepsy in females (OR = 2.22, 95% CI: 1.80–2.73). In the etiology analysis of epilepsy, those with structural (OR = 5.82, 95% CI: 3.92–8.64) and infectious (OR = 5.72, 95% CI: 3.93–8.32) epilepsy had a higher incidence of depression than those with epilepsy of unknown type. As the number of ASMs used increased, there was a tendency for the risk of depression to increase in female patients with epilepsy (≥ 2 ASMs: OR = 4.12, 95% CI: 2.11–8.05; 1 ASM: OR = 3.18, 95% CI: 1.65–6.13). Compared with oxcarbazepine, levetiracetam (OR = 2.47, 95% CI: 1.45–4.19) and topiramate (OR = 2.66, 95% CI: 1.34–5.30) increased the risk of depression, but sodium valproate (OR = 0.37, 95% CI: 0.21–0.65) reduced the risk of depression in females. Female patients with temporal area discharge (OR = 1.77, 95% CI: 1.37–2.30) were more likely to have depression than those without EEG discharge (Table 4).

**Table 4:** Binary logistic regression analysis for risk factors of depression among different sex in patients with epilepsy.

	Female				Male			
Variable			95%CI				95%CI	
	OR	*P*	Lower limit	Upper limit	OR	*P*	Lower limit	Upper limit
Age		**<0.001**				**<0.001**		
18–26	1.29	0.090	0.96	1.74	1.06	0.691	0.81	1.38
27–45	1.24	0.100	0.96	1.61	1.27	**0.036**	1.02	1.59
46–64	0.83	0.189	0.63	1.10	0.59	**<0.001**	0.46	0.75
≥ 65	1					1		
Ethnicity	–	–	–	–		**<0.001**		
Han Chinese	–	–	–	–	0.45	**<0.001**	0.32	0.62
Bai	–	–	–	–	0.22	**<0.001**	0.15	0.32
Yi	–	–	–	–	0.83	0.444	0.51	1.34
Tibetan	–	–	–	–	0.56	**0.035**	0.33	0.96
Lisu	–	–	–	–	0.63	0.066	0.39	1.03
others	–	–	–	–	1			
Occupation		**<0.001**				**<0.001**		
Farmer	0.59	**0.006**	0.40	0.86	0.92	0.725	0.57	1.48
Employee	0.31	**<0.001**	0.19	0.49	0.24	**<0.001**	0.13	0.42
Student	0.32	**<0.001**	0.21	0.48	0.31	**<0.001**	0.18	0.55
Others	1				1			
Years with epilepsy		**<0.001**				**<0.001**		
> 3 years	1.41	**0.003**	1.13	1.78	0.99	0.911	0.77	1.27
1-3 years	0.34	**<0.001**	0.25	0.45	2.51	**<0.001**	1.93	3.26
< 1 year	1				1			
Seizure frequency		**<0.001**				**<0.001**		
≥ 1 time per month	0.86	0.192	0.68	1.08	2.37	**<0.001**	1.74	3.23
< 1 time per month	0.36	**<0.001**	0.27	0.48	4.10	**<0.001**	3.07	5.48
Seizure-free	1				1			
Seizure type		**<0.001**				**<0.001**		
Focal	2.22	**<0.001**	1.80	2.73	10.53	**<0.001**	8.01	13.84
Generalized	1				1			
Etiology		**<0.001**				**<0.001**		
Structural	5.82	**<0.001**	3.92	8.64	1.59	**0.001**	1.20	2.10
Infectious	5.72	**<0.001**	3.93	8.32	1.41	**0.035**	1.02	1.93
Metabolic	1.30	0.198	0.87	1.96	9.65	**<0.001**	6.88	13.54
Immune	1.35	0.430	0.64	2.83	2.76	**0.008**	1.30	5.85
Unknown	1				1			
Number of ASMs used		**<0.001**				**<0.001**		
≥ 2	4.12	**<0.001**	2.11	8.05	0.63	**0.040**	0.41	0.98
1	3.18	**0.001**	1.65	6.13	1.37	0.133	0.91	2.06
0	1				1			
ASMs		**<0.001**				**<0.001**		
Sodium valproate	0.37	**<0.001**	0.21	0.65	0.46	0.046	0.22	0.98
Carbamazepine	0.58	**0.054**	0.33	1.01	0.53	0.103	0.24	1.14
Lacosamide	1.54	**0.198**	0.80	2.99	1.44	0.373	0.65	3.20
Lamotrigine	1.21	**0.571**	0.63	2.30	0.67	0.322	0.30	1.48
Levetiracetam	2.47	**0.001**	1.45	4.19	9.85	**<0.001**	4.42	21.96
Topiramate	2.66	**0.005**	1.34	5.30	1.87	0.157	0.79	4.46
Oxcarbazepine	1				1			
EEG findings		**<0.001**				**<0.001**		
Temporal area discharge	1.77	**<0.001**	1.37	2.30	1.61	**<0.001**	1.19	2.19
Others	0.42	**<0.001**		0.55	4.22	**0.002**	3.20	5.52
No discharge	1				1			

### 3.4 Factors influencing depression among adult male patients with epilepsy

Variables with *p* < 0.05 in the univariate analysis were included in the regression analysis, including age, ethnicity, occupation, years with epilepsy, seizure frequency, seizure type, etiology, number of ASMs used, ASMs, and EEG findings (Table 1). Age (*p <* 0.001), ethnicity (*p <* 0.001), occupation (*p <* 0.001), years with epilepsy (*p <* 0.001), seizure frequency (*p <* 0.001), seizure type (*p <* 0.001), etiology (*p <* 0.001), number of ASMs used (*p <* 0.001), ASMs (*p <* 0.001), and EEG findings (*p <* 0.001) had an independent effect on depression in male patients with epilepsy. People with epilepsy between the ages of 27 and 45 have a higher risk of depression (OR = 1.27, 95% CI: 1.02–1.59). Males with epilepsy for 1–3 years (OR = 2.51, 95% CI: 1.93–3.26) were more likely to develop depression than those with epilepsy for less than 1 year. Male patients with epilepsy with one seizure in a month (OR = 4.10, 95% CI: 3.07–5.48) had a higher probability of depression than those with no seizures in a month. Focal epilepsy was more likely to cause depression than generalized epilepsy in males (OR = 10.53, 95% CI: 8.01–13.84). In the analysis of epilepsy etiology, the prevalence of metabolic epilepsy was 9.65 times greater than that of cases classified as epilepsy of unknown etiology (OR = 9.65, 95% CI: 6.88–13.54). Compared with oxcarbazepine, levetiracetam (OR = 9.85, 95% CI: 4.42–21.96) significantly increased the risk of depression, but sodium valproate (OR = 0.46, 95% CI: 0.22–0.98) reduced the risk of depression in males. Male patients with temporal area discharge were 1.61 times (95% CI, 1.19–2.19) more likely to have depression than those without EEG discharge (Table 4).

## 4 Discussion

This study is the first large-scale investigation about the risk factors for depression among adult patients with epilepsy in Western China. A total of 3,620 patients with epilepsy are included in the study. There are related studies from Northeast, Northwest, and Sichuan regions of China (16, 32, 33), but they have smaller sample sizes and lack comparisons between different ethnicity. In the present study, the overall prevalence of comorbid depression among adults with epilepsy was 39.59% (26.74% in females and 12.85% in males). A meta-analysis reported the prevalence of depression in patients with epilepsy ranged from 4.5 to 59% (34). Another study showed that the prevalence ranged from 20 to 55% (35). In the present study, 39.59% of patients with epilepsy had depression, which was consistent with the findings of these previous studies. This result was lower than the rural prevalence of 52.6% and higher than the urban prevalence of 26.7% reported in studies from western China (20, 36). However, studies from the United Arab Emirates and West China Hospitals showed prevalence rates of 25 and 26.9%, which were lower than the 39.59% we reported (35, 37). The present study results indicated that females were at a higher risk of having depression than males, which was similar to the findings of studies from Ethiopia and India (38, 39). Our study indicated that female patients with epilepsy who considered depression had significantly higher PHQ-9 scores than males, which also suggested that depressive symptoms might be more severe in females compared males. The psychological problems of female epilepsy patients need to be paid more attention. One prospective study found an 11.28% prevalence of depression among male patients with epilepsy, which was consistent with our results (32). However, the prevalence of depression in women in this study was 18.66%, which was significantly lower than that shown by our study. One possible explanation is the difference in the diagnosis of depression and sample sizes. Another possibility is the region included in the present study (Dali, Yunnan Province, China) has many ethnic minorities, a low level of economy and education, and a lack of awareness of the disease. The higher risk of depression in females than in males may be attributed to different personalities and income inequalities (40).

Patients with epilepsy with lower levels of education were more likely to suffer from depression (41). Another study reported the opposite result (21). However, the present study did not find a correlation between the level of education of men and women and depression, which was in line with the results of Wang et al. (20). Place of residence was also considered a risk factor for depression in patients with epilepsy, as reported by a study from Ethiopia (21). They found that patients with epilepsy living in rural areas tended to be more prone to depression than those living in urban areas. However, we found that they lacked correlation. Therefore, more samples are required to analyze the condition. In the relationship between marital status and depression, we found no effect in patients with epilepsy of different sex, which was consistent with the findings reported by some studies (17, 25). On the contrary, other studies identified that being unmarried was an independent risk factor for epilepsy comorbid depression (32, 38). This interpretation may be related to differences in different countries, ethnicities, and sample sizes. At present, there was still a lack of research on the relationship between different ethnic groups and epilepsy comorbid depression. We found that male Bai people had a lower risk of depression, which might be related to the entertainment source of Bai people, such as dancing and singing. It was also conceivable that the Bai population might possess a reduced genetic predisposition to depression. However, the current body of research in this field remained insufficient. Nonetheless, in female epilepsy patients, susceptibility to depression showed no discernible association with ethnicity. Our analysis of different occupations revealed that office workers had the lowest risk of depression, and the trend was similar in females and males. This might be related to the higher income of office workers. However, there is still a lack of research on the occurrence of depression among different occupations in patients with epilepsy. More studies are needed on this topic. The relationship between age and depression in patients with epilepsy was controversial, some studies had suggested a relationship whereas others negated this view (42, 43). This study found that the relationship between age and depression was not obvious in female patients with epilepsy. Male patients with epilepsy aged 27–45 were 1.27 times more likely to suffer from depression than those aged over 65 years. However, male patients with epilepsy aged 46–64 years were less likely to develop depression than those aged over 65 years. A recent study had shown that patients with epilepsy aged 29–39 years show a higher tendency to depression (44). And this has similar results to our findings. A cross-sectional study of different ages and psychological stress showed that the PHQ-9 and PSS-10 scores were higher in the middle age group (26–44 years old) than high age group (>45 years old). It is possible that males between the ages of 27 and 45 may experience heightened psychological stress than others (45). This study also showed that patients with epilepsy were more likely to develop depression within 1–5 years, which was inversely correlated with the duration of the disease. Our results showed that males had the highest risk of depression within 3 years with epilepsy, whereas females were at risk after having epilepsy for more than 3 years, which was consistent with findings of the previous study. However, we did not study the condition over 5 years. Additional research is warranted to investigate the relationship between age and depression in patients with epilepsy.

One meta-analysis showed that more than 20 studies had reported that seizure frequency is a risk factor for depression (46). We also observed that seizure frequency was an independent risk factor for depression in both males and females. More frequent seizures might predict more severe depression (21). There may be a bidirectional relationship between depression and frequent seizures. One prospective study found that the occurrence of depressive symptoms was a predictor of epilepsy recurrence in adult patients with newly diagnosed epilepsy (47). Life-long mood disorders were also considered risk factors for seizure recurrence in adults (48). Moreover, focal seizures were more likely to present with depressive symptoms (49). Our findings also revealed that patients with focal epilepsy of both sexes were more prone to experiencing depression and significantly in males. This may be due to the existence of the same anatomical basis between depression and temporal lobe epilepsy. For example, patients with epilepsy and depression might have atrophy of the frontal lobe, hippocampus, and amygdala (50). Our study found a higher prevalence of depression in both females and males with temporal lobe discharge, which may explain the higher probability of depression in focal epilepsy.

In the analysis of the etiology of epilepsy, the prevalence of epilepsy of the unknown type was significantly higher other types. An epilepsy of unknown type was responsible for one-third of all etiologies (51), which could be attributed to the relatively less advanced diagnostic techniques for determining the underlying causes. Improving the diagnostic techniques remains pivotal for the categorization of epilepsy etiologies. This factor also contributed to the absence of genetic type data. Additionally, there was a higher proportion of female patients than male patients with epilepsy of an unknown type. This may potentially reflect sex discrimination within the diagnostic process. In the case of women, sex discrimination could lead to the development of depressive symptoms (52). The possibility of sex discrimination during the diagnostic process could also potentially contribute to the stigma experienced by women with epilepsy. However, there is a lack of research in this field. In the analysis of the relationship between the etiology of epilepsy and depression, we found that females with structural and infectious epilepsy and males with metabolic epilepsy had the greatest probability of depression, which might be potentially attributed to higher alcohol consumption among males. Infectious and structural etiologies were risk factors for suicidality in patients with epilepsy (53). Suicidal patients are often present with depressive symptoms. Our findings aligned with those of Lin et al. (53).

We also found that the number of ASMs used was an independent risk factor for depression in patients with epilepsy. A higher number of ASMs used often predicted a higher risk of depression. This was most pronounced in females, consistent with the findings of a previous study (54). It might also be related to the fact that some ASMs could cause mood disorders (55). Our study observed that sodium valproate decreased the risk of depression and levetiracetam increased the risk of depression in all patients with epilepsy. Topiramate increased the risk of depression in females, but not in male patients with epilepsy. Some studies indicated that sodium valproate, lamotrigine, and carbamazepine reduced the risk of depression in patients with epilepsy. Conversely, levetiracetam, benzodiazepines, and topiramate had been associated with an increased risk of depression in epilepsy patients, according to research findings (56–59). However, our results did not find an association between carbamazepine and depressive in patients with epilepsy. This may be due to the small sample size. Further research may be needed.

Nonetheless, our study has certain limitations. First, our study was a single-center, retrospective study; thus, there might be an under-representation of sample sizes. Second, we did not involve minors and only adult patients. Third, we did not exclude less common psychiatric disorders, such as bipolar disorder. Fourth, some patients had missing EEG findings (12.24%), which might have an impact on the analysis of EEG findings. Fifth, the diagnosis of depression was based on the patient’s PHQ-9 score at admission over the past 5 years rather than on a clinical diagnostic assessment, which may have led to an overestimation of prevalence. Sixth, as a concise depression assessment tool, the PHQ-9 did possess limitations and it might not comprehensively distinguish among patients experiencing anxiety.

## 5 Conclusion

Adult female patients with epilepsy have a higher susceptibility to depression than male patients with epilepsy. Furthermore, female and male patients with epilepsy have distinct risk factors for depression. Occupation, years with epilepsy, seizure frequency, seizure type, etiology, number of ASMs used, ASMs, and EEG findings are independent risk factors for depression in female patients with epilepsy. In male patients with epilepsy, age, ethnicity, occupation, years with epilepsy, seizure frequency, seizure type, etiology, number of ASMs used, ASMs, and EEG findings are independent risk factors for depression. Early detection and timely management of these risk factors may help reduce the incidence of depression in patients with epilepsy.

## Data availability statement

The raw data supporting the conclusions of this article will be made available by the authors, without undue reservation.

## Ethics statement

The research protocol received approval from the Ethics Committee of the First Affiliated Hospital of Dali University. This study is retrospective in nature and hence did not necessitate the signing of informed consent forms by the patients.

## Author contributions

WG: Data curation, Writing – original draft, Writing – review and editing. Y-xL: Methodology, Writing – original draft. YZ: Data curation, Methodology, Writing – review and editing. X-rL: Data curation, Methodology, Writing – review and editing. S-xW: Data curation, Methodology, Writing – review and editing. S-yZ: Data curation, Methodology, Writing – review and editing. E-sW: Data curation, Investigation, Writing – review and editing. X-jC: Data curation, Investigation, Writing – review and editing. YL: Conceptualization, Investigation, Project administration, Writing – review and editing.

## References

[1] Thijs RD Surges R O'Brien TJ Sander JW. Epilepsy in adults. Lancet. (2019) 393:689–701. doi: 10.1016/S0140-6736(18)32596-0 30686584

[2] Collaborators GBDE. Global, regional, and national burden of epilepsy, 1990-2016: a systematic analysis for the global burden of disease study 2016. Lancet Neurol. (2019) 18:357–75. doi: 10.1016/S1474-4422(18)30454-X, PMID: 30773428 PMC6416168

[3] The Lancet. Wanted: a global campaign against epilepsy. Lancet. (2012) 380:1121. doi: 10.1016/S0140-6736(12)61646-8, PMID: 23021266

[4] Mula M Kanner AM Jette N Sander JW. Psychiatric comorbidities in people with epilepsy. Neurol Clin Pract. (2021) 11:e112–20. doi: 10.1212/CPJ.0000000000000874, PMID: 33842079 PMC8032418

[5] Kanner AM. Depression in epilepsy: prevalence, clinical semiology, pathogenic mechanisms, and treatment. Biol Psychiatry. (2003) 54:388–98. doi: 10.1016/s0006-3223(03)00469-4, PMID: 12893113

[6] Hingray C McGonigal A Kotwas I Micoulaud-Franchi JA. The relationship between epilepsy and anxiety disorders. Curr Psychiatry Rep. (2019) 21:40. doi: 10.1007/s11920-019-1029-9 31037466

[7] Fiest KM Dykeman J Patten SB Wiebe S Kaplan GG Maxwell CJ. Depression in epilepsy: a systematic review and meta-analysis. Neurology. (2013) 80:590–9. doi: 10.1212/WNL.0b013e31827b1ae0, PMID: 23175727 PMC3589287

[8] Christian CA Reddy DS Maguire J Forcelli PA. Sex differences in the epilepsies and associated comorbidities: implications for use and development of pharmacotherapies. Pharmacol Rev. (2020) 72:767–800. doi: 10.1124/pr.119.017392, PMID: 32817274 PMC7495340

[9] Hermann B Seidenberg M Jones J. The neurobehavioural comorbidities of epilepsy: can a natural history be developed? Lancet Neurol. (2008) 7:151–60. doi: 10.1016/S1474-4422(08)70018-8, PMID: 18207113

[10] Singh T Goel RK. Epilepsy associated depression: An update on current scenario, suggested mechanisms, and opportunities. Neurochem Res. (2021) 46:1305–21. doi: 10.1007/s11064-021-03274-5, PMID: 33665775

[11] Kanner AM Saporta AS Kim DH Barry JJ Altalib H Omotola H. Mood and anxiety disorders and suicidality in patients with newly diagnosed focal epilepsy: An analysis of a complex comorbidity. Neurology. (2023) 100:e1123–34. doi: 10.1212/WNL.0000000000201671, PMID: 36539302 PMC10074468

[12] Altemus M Sarvaiya N Neill Epperson C. Sex differences in anxiety and depression clinical perspectives. Front Neuroendocrinol. (2014) 35:320–30. doi: 10.1016/j.yfrne.2014.05.004, PMID: 24887405 PMC4890708

[13] Gaus V Kiep H Holtkamp M Burkert S Kendel F. Gender differences in depression, but not in anxiety in people with epilepsy. Seizure. (2015) 32:37–42. doi: 10.1016/j.seizure.2015.07.012 26552559

[14] Zhu XR Zhu ZR Wang LX Zhao T Han X. Prevalence and risk factors for depression and anxiety in adult patients with epilepsy: Caregivers' anxiety and place of residence do mater. Epilepsy Behav. (2022) 129:108628. doi: 10.1016/j.yebeh.2022.108628, PMID: 35245762

[15] Bifftu BB Dachew BA Tiruneh BT Birhan Tebeje N. Depression among people with epilepsy in Northwest Ethiopia: a cross-sectional institution based study. BMC Res Notes. (2015) 8:585. doi: 10.1186/s13104-015-1515-z, PMID: 26482788 PMC4617742

[16] Liu Z Yin R Fan Z Fan H Wu H Shen B. Gender differences in associated and predictive factors of anxiety and depression in people with epilepsy. Front Psych. (2020) 11:670. doi: 10.3389/fpsyt.2020.00670, PMID: 32754069 PMC7365887

[17] Tegegne MT Mossie TB Awoke AA Assaye AM Gebrie BT Eshetu DA. Depression and anxiety disorder among epileptic people at Amanuel specialized mental hospital, Addis Ababa, Ethiopia. BMC Psychiatry. (2015) 15:210. doi: 10.1186/s12888-015-0589-4, PMID: 26328614 PMC4556015

[18] Chu C. Association between epilepsy and risk of depression: a meta-analysis. Psychiatry Res. (2022) 312:114531. doi: 10.1016/j.psychres.2022.114531, PMID: 35413534

[19] Munger Clary HM Snively BM Hamberger MJ. Anxiety is common and independently associated with clinical features of epilepsy. Epilepsy Behav. (2018) 85:64–71. doi: 10.1016/j.yebeh.2018.05.024, PMID: 29908386 PMC6093217

[20] Wang HJ Tan G Deng Y He J He YJ Zhou D. Prevalence and risk factors of depression and anxiety among patients with convulsive epilepsy in rural West China. Acta Neurol Scand. (2018) 138:541–7. doi: 10.1111/ane.13016, PMID: 30125939

[21] Addis B Wolde M Minyihun A Aschalew AY. Prevalence of depression and associated factors among patients with epilepsy at the University of Gondar Comprehensive Specialized Hospital, Northwest Ethiopia, 2019. PloS One. (2021) 16:e0257942. doi: 10.1371/journal.pone.0257942, PMID: 34695130 PMC8544874

[22] Dessie G Mulugeta H Leshargie CT Wagnew F Burrowes S. Depression among epileptic patients and its association with drug therapy in sub-Saharan Africa: a systematic review and meta-analysis. PloS One. (2019) 14:e0202613. doi: 10.1371/journal.pone.0202613, PMID: 30870423 PMC6417665

[23] Engidaw NA Bacha L Kenea A. Prevalence of depression and associated factors among epileptic patients at Ilu Ababore zone hospitals, south West Ethiopia, 2017: a cross-sectional study. Ann Gen Psychiatry. (2020) 19:19. doi: 10.1186/s12991-020-00268-5, PMID: 32174994 PMC7065310

[24] Kwon OY Park SP. Depression and anxiety in people with epilepsy. J Clin Neurol. (2014) 10:175–88. doi: 10.3988/jcn.2014.10.3.175, PMID: 25045369 PMC4101093

[25] Onwuekwe I Ekenze O Bzeala A Ejekwu J. Depression in patients with epilepsy: a study from Enugu, south East Nigeria. Ann Med Health Sci Res. (2012) 2:10–3. doi: 10.4103/2141-9248.96929, PMID: 23209983 PMC3507132

[26] Yang Y Yang M Shi Q Wang T Jiang M. Risk factors for depression in patients with epilepsy: a meta-analysis. Epilepsy Behav. (2020) 106:107030. doi: 10.1016/j.yebeh.2020.107030, PMID: 32248060

[27] Josephson CB Lowerison M Vallerand I Sajobi TT Patten S Jette N. Association of Depression and Treated Depression with Epilepsy and seizure outcomes: a multicohort analysis. JAMA Neurol. (2017) 74:533–9. doi: 10.1001/jamaneurol.2016.5042, PMID: 28241168 PMC5822203

[28] Viguera AC Fan Y Thompson NR Lapin B Chaitoff A Griffith SD. Prevalence and predictors of depression among patients with epilepsy, stroke, and multiple sclerosis using the Cleveland Clinic knowledge program within the neurological institute. Psychosomatics. (2018) 59:369–78. doi: 10.1016/j.psym.2017.12.003, PMID: 29580558

[29] Fisher RS Acevedo C Arzimanoglou A Bogacz A Cross JH Elger CE. ILAE official report: a practical clinical definition of epilepsy. Epilepsia. (2014) 55:475–82. doi: 10.1111/epi.12550, PMID: 24730690

[30] Fiest KM Patten SB Jette N. Screening for depression and anxiety in epilepsy. Neurol Clin. (2016) 34:351–61. doi: 10.1016/j.ncl.2015.11.003 27086983

[31] Fischer F Levis B Falk C Sun Y Ioannidis JPA Cuijpers P. Comparison of different scoring methods based on latent variable models of the PHQ-9: an individual participant data meta-analysis. Psychol Med. (2021) 52:3472–83. doi: 10.1017/S0033291721000131, PMID: 33612144 PMC9393567

[32] Li Q Chen D Zhu LN Wang HJ Xu D Tan G. Depression in people with epilepsy in West China: status, risk factors and treatment gap. Seizure. (2019) 66:86–92. doi: 10.1016/j.seizure.2019.02.014, PMID: 30818182

[33] Zhong R Chen Q Li M Li N Zhang X Lin W. Sex differences in anxiety in patients with epilepsy: status and risk factors analysis. Epilepsy Behav. (2021) 116:107801. doi: 10.1016/j.yebeh.2021.107801, PMID: 33578225

[34] Scott AJ Sharpe L Hunt C Gandy M. Anxiety and depressive disorders in people with epilepsy: a meta-analysis. Epilepsia. (2017) 58:973–82. doi: 10.1111/epi.13769, PMID: 28470748

[35] Alsaadi T El Hammasi K Shahrour TM Shakra M Turkawi L Almaskari B. Prevalence of depression and anxiety among patients with epilepsy attending the epilepsy clinic at sheikh Khalifa Medical City, UAE: a cross-sectional study. Epilepsy Behav. (2015) 52:194–9. doi: 10.1016/j.yebeh.2015.09.008 26448591

[36] Tong X An D Lan L Zhou X Zhang Q Xiao F. Validation of the Chinese version of the neurological disorders depression inventory for epilepsy (C-NDDI-E) in West China. Epilepsy Behav. (2015) 47:6–10. doi: 10.1016/j.yebeh.2015.03.012, PMID: 26004785

[37] Shen S Dong Z Zhang Q Xiao J Zhou D Li J. The overlapping relationship among depression, anxiety, and somatic symptom disorder and its impact on the quality of life of people with epilepsy. Ther Adv Neurol Disord. (2022) 15:17562864221138147. doi: 10.1177/17562864221138147, PMID: 36518552 PMC9742685

[38] Chaka A Awoke T Yohannis Z Ayano G Tareke M Abate A. Determinants of depression among people with epilepsy in Central Ethiopia. Ann Gen Psychiatry. (2018) 17:27. doi: 10.1186/s12991-018-0197-z, PMID: 29942342 PMC5998556

[39] Rashid H Katyal J Sood M Tripathi M. Depression in persons with epilepsy: a comparative study of different tools in Indian population. Epilepsy Behav. (2021) 115:107633. doi: 10.1016/j.yebeh.2020.107633, PMID: 33309426

[40] Zhong R Chen Q Li N Zhang X Lin W. Sex-based differences in the prevalence of and risk factors for depression in adult patients with epilepsy in Northeast China. Epilepsy Behav. (2021) 122:108201. doi: 10.1016/j.yebeh.2021.108201 34273741

[41] Pham T Sauro KM Patten SB Wiebe S Fiest KM Bulloch AGM. The prevalence of anxiety and associated factors in persons with epilepsy. Epilepsia. (2017) 58:e107–10. doi: 10.1111/epi.13817 28597927

[42] Ekinci O Titus JB Rodopman AA Berkem M Trevathan E. Depression and anxiety in children and adolescents with epilepsy: prevalence, risk factors, and treatment. Epilepsy Behav. (2009) 14:8–18. doi: 10.1016/j.yebeh.2008.08.015, PMID: 18804186

[43] Vujisic S Vodopic S Radulovic L Injac-Stevovic L. Psychiatric comorbidities among patients with epilepsy in Montenegro. Acta Clin Croat. (2014) 53:411–6. PMID: 25868308

[44] Bolling-Ladegaard E Dreier JW Kessing LV Budtz-Jorgensen E Lolk K Christensen J. Directionality of the association between epilepsy and depression: a Nationwide register-based cohort study. Neurology. (2023) 100:e932–42. doi: 10.1212/WNL.0000000000201542, PMID: 36414426 PMC9990426

[45] Zhang J Pan Y Hong J Guo H Wang M Liu X. Differences of medically unexplained symptoms among patients of different ages and sexes in the psychological clinic of a general hospital and the influencing factors of MUS: a cross-sectional study. Front Psych. (2022) 13:930212. doi: 10.3389/fpsyt.2022.930212, PMID: 35990083 PMC9386342

[46] Qin SK Yang ZX Guan ZW Zhang JH Ping X Lu Y. Exploring the association between epilepsy and depression: a systematic review and meta-analysis. PloS One. (2022) 17:e0278907. doi: 10.1371/journal.pone.0278907, PMID: 36520790 PMC9754200

[47] Zhong R Chen Q Zhang X Li N Lin W. Depressive and anxiety symptoms are predictors of seizure recurrence in adults with newly diagnosed epilepsy. Front Psych. (2021) 12:784737. doi: 10.3389/fpsyt.2021.784737, PMID: 34899438 PMC8653777

[48] Baldin E Hauser WA Pack A Hesdorffer DC. Stress is associated with an increased risk of recurrent seizures in adults. Epilepsia. (2017) 58:1037–46. doi: 10.1111/epi.13741, PMID: 28418198

[49] Peng WF Ding J Li X Mao LY Wang X. Clinical risk factors for depressive symptoms in patients with epilepsy. Acta Neurol Scand. (2014) 129:343–9. doi: 10.1111/ane.12191 24359278

[50] Kanner AM. Depression in epilepsy: a complex relation with unexpected consequences. Curr Opin Neurol. (2008) 21:190–4. doi: 10.1097/WCO.0b013e3282f4e978, PMID: 18317279

[51] Dubey D Alqallaf A Hays R Freeman M Chen K Ding K. Neurological autoantibody prevalence in epilepsy of unknown etiology. JAMA Neurol. (2017) 74:397–402. doi: 10.1001/jamaneurol.2016.5429, PMID: 28166327

[52] Stepanikova I Acharya S Abdalla S Baker E Klanova J Darmstadt GL. Gender discrimination and depressive symptoms among child-bearing women: ELSPAC-CZ cohort study. EClinicalMedicine. (2020) 20:100297. doi: 10.1016/j.eclinm.2020.100297, PMID: 32300743 PMC7152827

[53] Lin M Chen J Li S Qin Y Wang X Liu Y. Risk factors for suicidal tendency in people with epilepsy in China: a case-control study. Sci Rep. (2021) 11:2742. doi: 10.1038/s41598-021-81870-9, PMID: 33531579 PMC7854730

[54] Tombini M Assenza G Quintiliani L Ricci L Lanzone J Ulivi M. Depressive symptoms and difficulties in emotion regulation in adult patients with epilepsy: association with quality of life and stigma. Epilepsy Behav. (2020) 107:107073. doi: 10.1016/j.yebeh.2020.107073, PMID: 32320931

[55] Gangar K Bhatt LK. Therapeutic targets for the treatment of comorbidities associated with epilepsy. Curr Mol Pharmacol. (2020) 13:85–93. doi: 10.2174/1874467212666191203101606 31793425

[56] Hasegawa N Tohyama J. Positive and negative effects of perampanel treatment on psychiatric and behavioral symptoms in adult patients with epilepsy. Epilepsy Behav. (2021) 117:107515. doi: 10.1016/j.yebeh.2020.107515, PMID: 33610462

[57] Ho PH Leung WCY Leung IYH Chang RSK. Factors associated with depression in people with epilepsy: a retrospective case-control analysis. Hong Kong Med J. (2020) 26:311–7. doi: 10.12809/hkmj198310 32611830

[58] Joshi R Tripathi M Gupta P Goyal A Gupta YK. Depression in patients receiving pharmacotherapy for epilepsy: An audit in a tertiary care Centre. Pharmacol Rep. (2019) 71:848–54. doi: 10.1016/j.pharep.2019.04.021, PMID: 31398575

[59] Liu J Wang LN. Treatment of epilepsy for people with Alzheimer's disease. Cochrane Database Syst Rev. (2021) 2021:CD011922. doi: 10.1002/14651858.CD011922.pub4, PMID: 33973646 PMC8111487

